# Assessing and Improving the Use of Online Information About Child Development, Education, Health, and Well-Being in Low-Education, Low-Income Parents: Protocol for a Mixed-Methods Multiphase Study

**DOI:** 10.2196/resprot.9996

**Published:** 2018-11-20

**Authors:** Pierre Pluye, Christine Loignon, François Lagarde, Geneviève Doray, Reem El Sherif, Vera Granikov, Araceli Gonzalez Reyes, Mathieu Bujold, Roland Grad, Gillian Bartlett, Melanie Barwick, Tibor Schuster, Emmanuelle Turcotte, France Bouthillier

**Affiliations:** 1 Department of Family Medicine McGill University Montréal, QC Canada; 2 Département de médecine de famille Université de Sherbrooke Longueuil, QC Canada; 3 Fondation Lucie et André Chagnon Montréal, QC Canada; 4 Naître et grandir, Fondation Lucie et André Chagnon Montréal, QC Canada; 5 The Hospital for Sick Children Research Institute Toronto, ON Canada; 6 School of Information Studies McGill University Montréal, QC Canada

**Keywords:** consumer health information, information technology, primary health care, knowledge translation, literacy, vulnerable populations

## Abstract

**Background:**

This study is born from a partnership between Web editors of *Naître et grandir* (N&G) and *AboutKidsHealth* (AKH) and researchers who developed and validated the Information Assessment Method (IAM). N&G and AKH are popular Canadian websites with high-quality comprehensive information about child development, education, health, and well-being. IAM allows parents to assess online information and provide feedback to Web editors. High-quality online consumer health information improves knowledge, self-efficacy, and health. However, low-socioeconomic status (SES) parents underuse N&G and IAM, despite these parents being more likely to report decreased worries and increased confidence as outcomes from N&G information.

**Objective:**

The study is aimed to improve low-SES parents’ use of online child information and interaction with Web editors and explore subsequent health outcomes for parents and children.

**Methods:**

Multiphase mixed-methods design. Our general approach is centered on organizational participatory research. In phase 1, we will conduct a qualitative interpretive study to identify barriers and facilitators to using N&G information and to interacting with N&G editors via IAM; interview more than 10 low-SES parents about their experience with N&G and IAM and more than 10 nonusers of N&G and IAM; and use thematic analysis to identify main barriers and facilitators. In phase 2, we will integrate parents’ views (phase 1 findings) in N&G and IAM and implement a new version: IAM+N&G+. In phase 3, we will conduct a quantitative prospective longitudinal study (pre-/postimplementation monitoring of knowledge use and outcomes). We will compare the use of original (IAM and N&G) and new (IAM+ and N&G+) versions using Google Analytics variables, IAM variables, a material and social deprivation index, and demographics. We anticipate increased use post implementation (linear mixed modeling). In phase 4, we will conduct a qualitative descriptive study on outcomes of information use. We will interview more than 30 low-SES parents who receive and rate the N&G+ newsletter using IAM+ and analyze data in the form of life histories to describe how parents and children experience perceived outcomes.

**Results:**

The project was funded in 2017 by the Canadian Institutes of Health Research and received an ethics approval by the McGill University’s institutional review board. Data collection for phase 1 was completed in 2018. Phases 2 to 4 will be conducted until 2020. Findings from this study will also be used to develop a free toolkit, useful to all Web editors, with recommendations for improving health information for low-SES persons and interactions with them using IAM.

**Conclusions:**

The results of this study will provide a deep understanding of how low-SES parents use online child information and interact with Web editors. Following the implementation of IAM+N&G+, results will also elucidate subsequent health outcomes for low-SES parents and children after interaction with Web editors has been optimized.

**International Registered Report Identifier (IRRID):**

PRR1-10.2196/9996

## Introduction

### Rationale

Early life experience is a determinant of social inequalities in physical, mental, and social well-being [1-6]. Children living in poverty are more likely to suffer from developmental and health problems, and childhood interventions can decrease incidence and prevalence of these problems [7-12]. The proposed project is aimed to assess and improve a childhood intervention: the *Naître et grandir* (N&G) website and newsletter on child development, education, health, and well-being, which includes the Information Assessment Method (IAM) that allows N&G readers to continuously assess, and subsequently the N&G editors to improve, the content shared on the N&G website and newsletter.

Parents with low education and low income, hereafter referred to as parents with low socioeconomic status (SES), typically have a low literacy level (limited ability to acquire, understand, evaluate, and use written information). Low parental literacy level is particularly detrimental to child health: a low literacy level is associated with worse health status, difficulties accessing health care, and poorer preventive health behavior and self-management of health problems [13-23].

Education and income are the most important SES indicators, and together, they are strongly associated with child health status [24,25]. According to research on information-seeking behavior, parents with low SES have greater information needs than parents with high SES [26]. The use of high-quality online information can improve quality of life and have positive family, economic, and social impacts on low-SES parents, including refugee and homeless parents [27-31]. High-quality information and literacy-related interventions can reduce unnecessary calls and visits to health professionals, increase knowledge and self-efficacy, and improve health [16-19,32-47].

N&G [48] is a website independent from industry funding that provides high-quality information (based on research syntheses and validated by experts) on child development, education, health, and well-being. N&G users can access hundreds of information pages (1 page per topic) that are organized in age group categories, ranging from pregnancy to the age of 8 years. N&G also produces a weekly newsletter to support parents, including those with a low literacy level, having children under the age of 8 years.

N&G partnered with investigators from McGill University to validate and implement the IAM to continuously assess and improve content shared on the N&G website and newsletter [49-53]. In line with the Canadian Institutes of Health Research (CIHR)’s definitions [54], the IAM is a knowledge translation tool for monitoring N&G information use, and its impact on parents is measured by expected health/well-being benefits. It is also fostering parent engagement by enabling parents to interact with N&G editors by providing feedback on the information content. The IAM questionnaire includes 7 questions (with clickable answers and 2 comment boxes), allowing users to rate on the situational relevance, cognitive/affective impact, intention to use and expected benefits of specific information content (N&G information page), and write comments.

**Figure 1:**
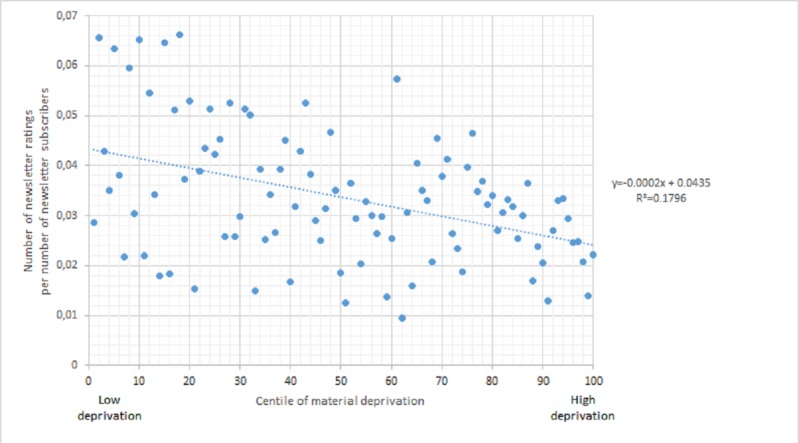
Social gradient in assessing *Naître et grandir* (N&G) information with Information Assessment Method (IAM). A dot represents the total number of IAM ratings completed by all N&G newsletter subscribers living within the postal code areas of a Canadian centile of Material Deprivation. There is a negative linear relationship as areas with higher deprivation have a lower proportion of newsletter ratings/subscribers. The correlation coefficient is −.42 (*P*<.001).

During our pilot phase (September 1, 2014, to August 31, 2016), we collected 34,021 IAM ratings (completed IAM questionnaires) from parents, relatives, and professionals (education, health, and social services) who read N&G content. In line with studies on social inequalities in Web information use [55-61], the statistical analysis of these ratings revealed a social gradient: low-SES parents underuse N&G and the IAM (Figure 1). Results also indicated that low-SES parents are more likely to report decreased worries and increased confidence as a result of using N&G information [50]. There is a need to understand this gradient to improve N&G content and reach low-SES parents and a need to explore how the use of knowledge translates into health and well-being outcomes for low-SES parents and children.

### Goal and Objectives

This study (research protocol) has been recently funded by CIHR. As per CIHR’s definition of knowledge translation (“making people aware of knowledge and facilitating knowledge use to improve health”) [62], our goal is to improve how low-SES parents engage with and use online information about child development, education, health, and well-being; learn how this format for knowledge sharing translates to improved health and well-being outcomes for low-SES parents and their children; and explore their interaction with Web editors to influence content (responsible for websites’ content). On the basis of the N&G team's (the director and 2 coordinators) questions and using the Knowledge-to-Action framework [63], we determined 4 research objectives:

- Objective 1: Identify low-SES parents’ views on barriers and facilitators to accessing, understanding, and using N&G information content and to interacting with N&G editors via the IAM.
- Objective 2: Adapt N&G content/functions and the IAM tool (user-centered design) to reduce barriers and optimize facilitators identified in phase 1 and implement new versions labeled N&G+ and IAM+.
- Objective 3: Evaluate whether N&G+ and IAM+ result in a higher proportion of low-SES parents engaging with the N&G site and the content provided and interacting with editors via the IAM.
- Objective 4: Describe how expected health and well-being benefits of N&G+ information use (reported via IAM+) are experienced by parents with low SES and their children.

### Systematic Literature Review and Theoretical Model

**Figure 2:**
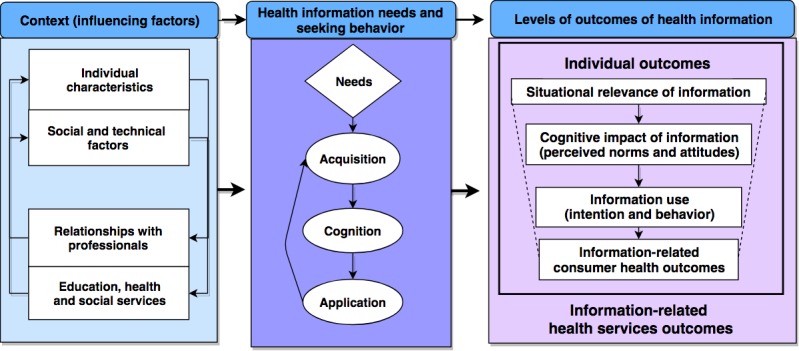
Theoretical model.

The nominated principal investigator (NPI) led a CIHR-funded systematic review and proposed a comprehensive harmonized typology of (1) outcomes associated with the use of online consumer health information and (2) conditions (network and resources) leading to these outcomes [64-67]. A total of 68 studies were analyzed using framework synthesis [68,69]. This synthesis was based on an initial framework derived from information studies [49,70-78] and led to propose an innovative theoretical model (Figure 2) [66,67]. This model includes positive and negative health outcomes of online consumer health information in a primary care context and is (information) consumer oriented.

The model comprises 13 main concepts (42 factors and outcomes) and will be used in this study to inform data collection and analysis, for example, the phase 1 qualitative interview guide and interpretive thematic analysis. The model will contribute to establish a chain of evidence linking ultimate outcomes of information (such as health outcomes), intermediary outcomes (such as cognitive impact of information), and conditions associated with outcomes (information needs and seeking behaviors and contextual factors).

#### Outcomes

The model includes an organizational level of information outcome (eg, increased or decreased use of health services) and 4 individual levels of outcomes of information. The latter outcomes reflect how information is valuable from the consumer’s perspective: situational relevance, cognitive impact, use of information (conceptual, legitimating, instrumental, and symbolic use), and subsequent health and well-being outcomes.

#### Conditions

The model includes conditions associated with outcomes of information in relation to a specific situation: a particular information object is acquired or delivered (eg, a Web page or a newsletter) in a particular situation (eg, before or during or after an encounter with someone) directly or with help from a relative or a professional. The main conditions are information needs and seeking behaviors; individual characteristics (such as electronic health literacy); social and technical factors (such as social networks); relationships with professionals (such as teachers, clinicians, and social workers); and access to education, health, and social services. The first phase of this study will specifically look into conditions experienced by low-SES parents, such as media competence.

Direct acquisition of online information depends on an impetus to search (motivation), momentary internet connection, internet search skills, and one’s ability to understand the content that may or may not be adapted to their individual literacy level [79-81]. Approximately 95% of parents of preschool children have direct individual access to the internet in Quebec [61], and parents can also access it at office workplaces and public libraries [79-81]. Literacy level is generally defined as the degree to which a person has the ability to “acquire, understand, evaluate, and use information” needed to obtain services and make appropriate decisions [23]. Computer literacy, information literacy, and health literacy are interdependent (eg, a person with a low literacy level has a low level of health literacy). Culture is central in literacy, and one’s literacy level depends on one’s ability to understand systems of symbols from one’s own culture or a dominant culture and language, for example, immigrants and refugees may have a higher literacy level in their country of origin compared with their adoptive country [23]. As mentioned in our model, the literacy level is *situational* and *contextual*, given that a social network can compensate for an individual’s low literacy level.

Mediated (by someone else) acquisition of information is very common. The absence of an individual connection 24/7 is no longer the primary barrier to seeking health information [80]; for example, our pilot data show that 18.1% of IAM ratings concerned N&G information seeking for someone else’s child (eg, relative’s, friend’s, neighbor’s, or client’s child). Even homeless parents or recent immigrants and refugees can acquire online consumer health information directly or mediated by their social network, including community organizations; public libraries; and education, health, and social services [23,27-31]. Indeed, information studies show that consumers combine mediated information with direct acquisition of online health information, the latter allowing them to probe information provided by professionals [64-67]. For analytical purpose, we conceive these combinations as the interpenetration of social systems centered on communicative action, for example, the health system (mediated access) and a consumer parent system (direct access and mediated access via their social network) [82-85].

### Significance

Our project focuses on all Canadian parents with low SES who acquire (direct or mediated) online information about child development, education, health, and well-being. In Canada, 42% of nonelderly adults [16,17] and 15% of children live in low-income households [86]. In Quebec, 36% of parents report living in poverty [87]. The demand for information is very high, resulting in frequent internet searches; for example, parents search the internet for child-related information on average 1.3 times per week in Quebec, and 73% of parents report that the internet constitutes their first-line source of parenting information [88].

**Table 1:** Main features of *Naître et grandir* and *AboutKidsHealth*.

Main features	*Naître et grandir* [48]	*AboutKidsHealth* [94]
Website annual traffic	24 million worldwide visits	16.8 million worldwide visits
Information	Developmental, educational, health, and well-being information about children aged under 8 years	Health information about children aged under 19 years
Language	French	English, French, and 10 other languages
Targeted audience	Parents, relatives, and caregivers	Parents, relatives, and caregivers and health professionals

Specifically, our project is important for about half the population of parents of children under 19 years, namely, parents with a low literacy level. Low literacy is a major concern in Canada [16,17,19,20,23]. Results of the 2011 to 2012 survey of a representative sample of 25,267 Canadian parents aged 16 to 65 years showed that 49% of parents have a low literacy level: [89,90] they have difficulty finding, understanding, and using information presented in a dense or lengthy text; navigating complex digital texts; interpreting and evaluating information (constructing meaning); and disregarding irrelevant or inappropriate content when there is competing information (including when correct content is more prominent). Furthermore, our results may help any parent who faces a transitory low literacy level because of a stressful situation. This interdependence between information and emotion is well established in the information literature [91,92].

Our project nevertheless does not focus on the few *information-poor* parents without access (direct or mediated) to online information. In the United States, only 1% of 18- to 29-year olds did not use the internet in 2015 [93]. The Quebec 2015 survey of a representative sample of 23,693 parents of preschool children showed that only 1.5% of parents never know where to find child information [61]. According to information studies, the *information-poor* parents (1) perceive themselves as persons who cannot be helped, (2) adopt self- or group-protective behaviors, (3) are secretive and mistrust others, and (4) consider exposure to information as a risk (harm outweighing benefits) [30].

Our project engages a partnership between N&G and *AboutKidsHealth* (AKH) [94], both popular comprehensive Web-based resources (Table 1). Both resources contain text with a readability level below high school (this level may be higher for disease-related information), visual illustrations, and complementary simple illustrative videos. For each Web page, an audio help system highlights words as they are spoken. In 2016, our librarian did an environmental scan of Canadian parental websites and found multiple specialized resources but only 12 comprehensive resources (including N&G and AKH).

Although this study is a priority in a Canadian context, our results can be important to and applied by all Web editors who provide health information, thereby benefitting all adults with a low literacy level (not only parents). For example, Health Canada policies recommend funding projects that aim to improve access to, and conduct research on, information for Canadians with a low literacy level [23]. Information systems are key components of health systems and crucial to meet human rights and the democratic right to know and communicate [31,95-100]. Among adults, the most frequent searches for information are about health [101]. The demand for high-quality low-literacy health information is, hence, very high.

## Methods

### Organizational Participatory Research Approach

Our project uses an organizational participatory research (OPR) approach. OPR is a form of integrated knowledge translation that blends action research and organizational learning to undertake research with organizations and improve practice [102-107]. N&G (organization) proposed the research questions and will participate in all research steps. The NPI has OPR experience and expertise. The NPI led a CIHR-funded systematic review on OPR key processes and outcomes [107]. A steering committee composed of the NPI, the coprincipal investigator, the partner (principal knowledge user), another knowledge user (AKH), and 2 low-SES parents will meet before and after each phase for planning and interpreting results, respectively. All team members will be consulted and have the opportunity to influence the steering committee’s decision making.

### Methodology and Methods

A mixed-methods multiphase design will be used [108,109]. Ethical approval has been recently obtained from the institutional review board (IRB) of the Faculty of Medicine at McGill University (IRB#A10-E69-17B). Phases 1, 3, and 4 will be informed by our theoretical model (Figure 2).

#### Phase 1 (Objective 1): Identify Barriers and Facilitators to Use Naître et Grandir and Interact With Editors Via Information Assessment Method

##### Design

We will conduct an exploratory qualitative interpretive study to have a better understanding of barriers and facilitators in using N&G and interacting with editors via IAM [110]. Qualitative research is appropriate as it provides in-depth descriptions from the stakeholders’ viewpoint and helps researchers to understand their constitutive elements and variants [111].

##### Setting and Participants

Participants will be 20 users and 20 nonusers of IAM and N&G (additional participants will be recruited to achieve data saturation). First, users will be recruited among low-SES parents who indicate that they agree to be contacted when they complete an IAM questionnaire on N&G and who have used N&G and IAM at least once. N&G will send the group an invitation to participate in a research project via an email containing the research team’s full identification and contact information. The research team will only communicate with N&G users who responded with an interest to participate in the research project via email or phone. Eligible parents will have no high school diploma and have an annual family income lower than Can $40,000 (Quebec poverty line). Second, nonusers (10 who never used the IAM and 10 who never used N&G) will be recruited by a principal investigator (CL) and a collaborator (GD, N&G Director) who work with community organizations for parents with low SES.

##### Data Collection

A research assistant having experience in qualitative interviews with low-SES persons will conduct individual 60-min semistructured face-to-face interviews at the location of each participant’s choice. In line with sociological and information studies [112,113], interviews will explore how participants experience, conceptualize, perceive, and understand aspects of N&G information and the IAM. The interview guide will consist of open-ended questions addressing the participants’ routines in terms of internet use (both for their personal and children’s development information needs), their experience in the use of IAM and N&G’s information, the facilitating aspects of N&G and IAM, perceived barriers in their experience, and suggestions to improve the N&G website and IAM. On the basis of the theoretical model, the interview guide will be developed in a simplified language with input from team members and the steering committee. We will conduct 2 pilot interviews to ensure the questions are comprehensible for the participants. The recruited respondents will receive Can $20 as compensation for their time. In addition to the research assistant’s observation notes, interviews will be audio recorded and transcribed verbatim.

##### Data Analysis

Two principal investigators (PP and CL) have experience in qualitative research and 1 (CL) has experience in qualitative research with populations in situations of vulnerability. With the research assistant and a research trainee, they will read notes and interviews and meet regularly to build memos (meeting minutes with arguments for and against each analytical decision, as rigor is mainly based on researchers’ reflexivity in this design [110]), case summaries, and themes (definitions and key examples) using hybrid deductive-inductive thematic analysis [114,115]. The research assistant will manage the analysis using specialized software (NVivo11). Themes will be derived from our theoretical model (Figure 2; deductive coding) and emerge from the data (inductive coding). Major themes will be barriers and facilitators. For each type of participant (user/nonuser), additional parents will be recruited up to saturation (no new parents’ views of barrier or facilitator). Then, we will harmonize themes [116]. For each theme and definition, we will identify terms, confirm the usage of these terms in reference documents, distinguish correct from incorrect usage, and retain terms that facilitate unambiguous communication. This will lead us to classify, group, and clarify barriers and facilitators in a coherent taxonomy, which will be reviewed by team members and the steering committee. Findings will provide recommendations for improving IAM and N&G.

#### Phase 2 (Objective 2): Improve Naître et Grandir and Information Assessment Method

Two team members (FL and GD) are head directors of the N&G website and will lead this phase. Phase 1 findings will inform the production and implementation of IAM+N&G+ (user-centered design). This version will integrate the perspectives of low-SES parents, the steering committee, research team members, and N&G staff on how to overcome barriers and optimize facilitators. Production of this version will be planned with 2 Web editors and 2 Web engineers from the N&G team. According to Web engineers, 6 months is an ample time frame for production and beta testing. All necessary resources at N&G will be made available to implement IAM+N&G+. Two half-day meetings with principal investigators and N&G staff have been deemed sufficient to plan changes with editors and engineers. The N&G Director stated that she will implement what will be requested by the phase 1 participants, as her mandate (N&G mission) is to specifically address information needs of parents with low SES. For their part, AKH will redesign their website, integrate phase 1 results in this study, and implement IAM+.

#### Phase 3 (Objective 3): Evaluate Use of Information Assessment Method+ Naître et Grandir+, Information Use, and Benefits

##### Design

We will conduct a quantitative prospective longitudinal study to evaluate the impact of the intervention (IAM+N&G+).

##### Setting

Weekly numbers of N&G sessions and IAM ratings will be monitored over 2 years. A session starts when someone opens a Web page and ends when the person (or a relative with the same IP address) does not use the website for more than 30 min. For each quintile of SES, data will be collected for 9 months preimplementation (IAM/N&G) and 9 months postimplementation (IAM+N&G+). To avoid bias related to the *novelty effect*, data collected in the 3 months immediately following the implementation will be excluded from the analysis. The chosen periods also ensure seasonal comparability of the collected outcome data (before and after intervention).

##### Participants

All N&G readers and IAM raters across Canada will participate.

##### Measurement

N&G and McGill already use Google Analytics, an objective and reliable automatic data collection of N&G readers’ demographic characteristics and website use behavior (based on javascript codes in each Web page) [117-120]. IAM raters’ demographic data are collected with a questionnaire (linked to an anonymized identifier). The validated IAM questionnaire [53] collects self-reported information use and subsequent expected health/well-being benefits for parents and their child.

**Table 2:** Types of outcomes monitored weekly.

Type	Outcome	Description and data source
A1	N&G^a^ sessions	Weekly number of unique sessions (Google Analytics)
A2	N&G read	Weekly proportion of sessions with at least 1 page entirely read (Google Analytics)
A3	IAM^b^ ratings	Weekly number of IAM questionnaires submitted
B1	N&G mediated	Weekly proportion of IAM ratings information used for the child of someone else
B2	N&G used	Weekly proportion of IAM ratings information used for oneself and one’s child
C	Expected benefits	Weekly proportion of IAM ratings expected health or well-being benefit for a parent and child, including at least 1 of the following self-perceived benefits (IAM choices of response): improvement of the health or well-being of a child, being less worried, prevention of a problem or the worsening of a problem, handling a problem, and being more confident to decide something with someone else

^a^N&G: *Naître et grandir*.

^b^IAM: Information Assessment Method.

##### Outcomes

In line with CIHR knowledge translation guidance [54], 3 types of outcomes will be considered weekly (Table 2): (A) N&G and IAM use, (B) self-reported N&G information use, and (C) subsequent expected benefits.

##### Covariates

The main predictor will be participants’ SES as determined by the Quebec Index of Material and Social Deprivation, a validated ecological measure of the (education, income, and employment) disadvantage of a given geographic area (postal code) [121,122]. For each session, an index will be automatically assigned using (1) the reported postal code and the Canadian Deprivation Index Assignment Program (CDIAP) of the Public Health Agency of Canada when the data source is IAM and (2) the postal code matched to the session geotag via the “Dissemination Area Boundary File” of Statistics Canada and the CDIAP when the data source is Google Analytics. This will allow for the identification of low-SES participants (highest quintile of material and social deprivation). Using Google Analytics, participants’ demographic variables that will be considered in all analyses are age, gender, rural/urban location, and province. In addition, Web page–specific variables will be included to assess potential confounding and effect modifications. For each session, the type of device (Google Analytics: phone, tablet or laptop, and desktop), the audio-guide use (Google Analytics: yes or no), and the readability score of pages entirely read (automatic extraction and measurement using a text classifier validated for French) [123] will be collected.

##### Study Size

On the basis of our 2-year pilot data (2014/09-2016/08), we anticipate about 5 million N&G sessions and 15,000 IAM ratings from Canadian participants during each period (pre- and postimplementation).

##### Anticipated Results

The IAM+N&G+ will result in an increase of all outcomes (N&G and IAM use, self-reported N&G information use, and subsequent expected health and well-being benefits for parents and children such as *decreased worries* and *health improvement*, respectively).

##### Statistical Analysis

Linear mixed modeling will integrate spatial analytics (geomatics) and account for the clustered nature of the data. The pre/post status, the SES quintile of deprivation, and potentially confounding variables will be included as fixed-effects covariates. The inference model will incorporate random effect terms for individual variables and Web page–specific variables. For each outcome, an interaction term of the SES quintile of deprivation and the pre/post status will be included to assess the pre/post change for each quintile. Estimated regression coefficients and variance parameters will be reported along with appropriate confidence intervals.

#### Phase 4 (Objective 4): Describe Parent and Child Outcomes of Naître et Grandir+ Information Use

##### Design

We will conduct a qualitative interpretive study to generate an in-depth description of parent and child outcomes from the parents’ perspective [124]. On the basis of our systematic review [66,67], we anticipate 3 types of parent outcomes (decreased worries, increased confidence, and self-management) and 2 types of child outcomes (prevented problem and improved development, health, and well-being). Our pilot data (34,021 IAM ratings) also suggest parents rarely report potential negative consequences of using information, for example, vaccine adverse effect (n=183; 0.5%). In outcomes research, qualitative methods are appropriate for exploring complex outcomes from the stakeholders’ perspective, such as life experiences [111,125-131]. In our study, information can influence parent decision making, but the relationship between information and decision is not simple. Our qualitative study will (1) identify causal events, (2) map them in a complex causal network, and (3) build a chronological chain of qualitative evidence between N&G information and a parents’ decision that affects knowledge, attitude, or behavior [131,132].

##### Participants

Participants who received the N&G+ newsletter, used IAM+ at least once, have agreed to be contacted for research, and live in a high deprivation area will be approached for recruitment. The recruitment procedure will be the same as for phase 1. In 2015, more than 24,000 families received, upon request, the weekly newsletter. We will begin by recruiting 30 parents (and continue recruitment, as needed, to achieve data saturation) who report, via IAM+, positive health and well-being outcomes and/or potential negative consequences of information (prioritizing those who live in the areas of highest deprivation). As we are looking for individual stories for about 6 types of outcomes (some less frequently reported than others), data saturation may not be reached with fewer than 50 participants [111,133-135].

##### Data Collection

The research assistant having experience in qualitative interviews with low-SES persons will conduct 60-min face-to-face interviews to elicit participants’ IAM+ ratings. The interview guide will be based on our theoretical model (Figure 2) and input from team members and the steering committee. To stimulate recall (memory), the interviewee will be given the list of recent texts they rated and their ratings. For each newsletter, the research assistant will ask open questions regarding what happened to them and/or their children that led them to report an expected outcome (positive or negative). Participants will be interviewed twice 3 months apart to increase the number of described outcomes and avoid fatigue of long interviews. Participants will receive Can $20 per interview as compensation for their time. In addition to the research assistant’s observation notes, interviews will be audio recorded and transcribed verbatim.

##### Data Analysis

Two principal investigators (PP and CL) have experience in qualitative research. With the research assistant, a collaborator anthropologist having expertise in life histories (MB) and a research trainee will read notes and interviews and meet regularly. For each case (information used with at least 1 outcome experienced by a parent and a child), they will interpret the data in the form of a *small story* [112,136-138]. This method allows researchers to describe a person’s individual experience (including all perceived influences) and helps the researcher understand the individual’s attitude and behavior [33]. In research meetings, 2 questions will be answered through recorded discussion of arguments for and against each analytical decision (rigor based on sharing reflexivity): is the case story clear? and are the causal network and chain of evidence trustworthy? Disagreements about clarity and trustworthiness of case stories will be resolved with 3 other team members having experience in qualitative research (Bouthillier, Thoër, and Smythe). Case stories will be reviewed by all team members and the steering committee. This may detect issues and result in the principal investigators and research assistant revising parts of their analysis.

##### Anticipated Results

This will generate up to 10 case stories per outcome type, being the first in-depth qualitative description of low-SES parents’ perspective on outcomes of online child information use.

## Results

The project was funded in 2017 by the CIHR and received an ethics approval by the McGill University’s IRB. Data collection for phase 1 was completed in 2018. Phases 2 to 4 will be completed by 2020. Findings from this study will be used to develop a free toolkit, useful to all Web editors, with recommendations for improving health information for low-SES persons and interactions with them using IAM. Results will be published in peer-reviewed journals and presented at national and international scientific conferences.

## Discussion

### Direct Impact on *Naître et grandir* and *AboutKidsHealth*

Any improvement of knowledge translation tools and websites such as IAM, N&G, and AKH can have an important impact as it affects a large population of individuals with a low literacy level. In fact, the effectiveness of childhood education interventions has been demonstrated repeatedly [16-19,23,27-47]. In our 2-year pilot data, parents expected health and well-being benefits (for themselves or their child) from using N&G information in 65.4% of all IAM ratings (n=34,021) [50]. In accordance with knowledge translation and implementation research [62], we will not replicate effectiveness studies and rather focus on improving interventions that work.

Our project can improve engagement (number of visits) with websites providing high-quality low literacy information; in turn, online parenting information improves parents’ knowledge, attitudes, and behaviors [139-145]. Given our integrated knowledge translation (participatory research) approach, N&G and AKH will adapt their content for low-SES users as we generate results. In addition, IAM+ will better support low-SES parents’ interactions with Web editors and empowerment. Thus, our results can immediately benefit millions of information users with a low literacy level (children’s parents and relatives), for example, in Canada (Table 3).

IAM+N&G+ can be seen as an innovative intervention that complements traditional literacy programs (eg, family literacy classes). Indeed, interventions that somewhat compensate for a low literacy level can greatly improve parents’ and children’s health and well-being [23]. Thus, our results can have a positive impact on 55% of the Canadian working age, those who have a low level of *health literacy* and need compensatory help to manage their health [23,89,90].

Specifically, IAM+N&G+ can help Canadians in francophone minority communities, as 500,000 annual N&G website visits originate from them. Research with these communities is very underfunded, thus a CIHR priority [146]. As stated in the October 2016 report of the Commissioner of Official Languages [147], early childhood development in these communities *is hindered by a lack of resources*, and *the absence of specific funding* has left them *vulnerable and often incapable of meeting their needs*.

**Table 3:** Targeted population in Canada.

Across Canada	IAM^a^ *Naître et grandir* (new version)	IAM *AboutKidsHealth* (new version)
Families^b^	1.3 million families have French as mother tongue (couples with children and single-parent families with at least 1 child)	5.8 million families (couples with children and single-parent families with at least 1 child)—all languages
Children’s parents and relatives^b^	2.8 million adults aged 20 to 69 years have a low literacy level (and French as mother tongue)	12 million adults aged 20 to 69 years have a low literacy level
Website visits from Canada	8.3 million^c^ visits in 2015 mainly from Quebec (7.8 million), Ontario (304,000), and New Brunswick (70,000)	1.5 million visits in 2015 mainly on Web pages in English (58%), French (16%), Spanish (14%), and Arabic (5%)

^a^IAM: Information Assessment Method.

^b^Statistics Canada 2015.

^c^76% of parents of children aged under 8 years consult *Naître et grandir* in Quebec (on average 1.3 times per week).

### Potential Impact Beyond *Naître et grandir* and *AboutKidsHealth*

Results will inform the development of an open access online tool kit on how to adapt websites and IAM+ for a low literacy audience (a growing consideration of many Web editors). The tool kit will be designed to be generalizable to all types of online consumer health information in Canada. It will include guidance with 3 main messages: (1) how to produce simple information in lay language with audio and visual content, (2) how to better interact with low-SES persons using IAM+, and (3) how to use consumers’ IAM+ ratings and feedback to continuously optimize information content. The tool kit will be freely available to any Web editor via the Quebec SPOR-SUPPORT Unit and IAM websites. The NPI has experience in developing tools and leads the method development platform of the Quebec SPOR- SUPPORT Unit.

Our primary knowledge translation goal for knowledge user audiences will be to raise awareness about our main messages and drive attention to the tool kit. A secondary knowledge translation goal will be to support the implementation of the toolkit by Web editors. Knowledge users will disseminate our work in multiple Canadian organizations. As per reviews on scaling up [148-150], we will update our 2016 environmental scan and contact all Canadian websites targeting parents. A specialized librarian (collaborator) will reach additional health websites through librarian listservs and peer networks. Team members will raise general awareness for the tool kit and deliver our main messages to a variety of academic and nonacademic knowledge user audiences via a range of knowledge translation strategies (conference presentations, open access peer-reviewed publications, plain language summaries and grey literature, social media, and networks). Multiple knowledge translation channels will be used including Twitter (@UniteSoutien) and websites such as the Quebec SPOR-SUPPORT Unit and the IAM websites [151,152].

### Contribution to Scientific Knowledge

Our project will advance knowledge on the value of online information for, and interaction with, low-SES persons to strengthen health systems. On the one hand, this project is the first to systematically explore health and well-being outcomes for low-SES parents and children associated with parenting information websites. Most studies focus on discussion forums that can be intimidating to low-SES parents [140,142,143,153,154]. On the other hand, many studies concern relational marketing and website feedback buttons [52,155-158], but we know of none that addresses interactions between low-SES persons and Web editors (such as interaction through IAM+).

### Timeline and Potential Challenges

**Figure 3:**
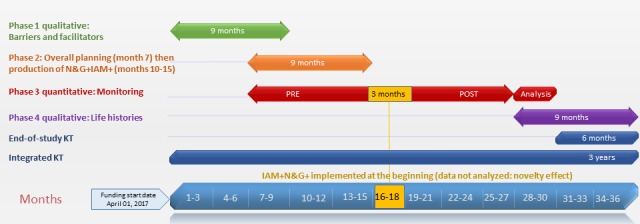
Study timeline. N&G: *Naître et grandir*; KT: knowledge translation; IAM: Information Assessment Method.

Overall, 3 years will be needed to complete this project (Figure 3). Our phases are simple and well defined, and our planning is realistic. In phases 1 and 4, we anticipate no recruitment difficulties. Many parents have already agreed to be contacted for research, and N&G will facilitate the recruitment. Phase 2 is feasible, and the deliverables are realistic given that N&G has already implemented IAM, McGill and N&G have a strong OPR partnership, and N&G is committed to implementing IAM+N&G+. In phase 3, the large number of N&G readers guarantee a large sample. To control for potential seasonal variation in the number of sessions pre- and postimplementation, monitoring will be conducted during the same seasons. To control for contextual changes, variables such as type of device, age, gender, and location are covariates. We have opted for a prospective longitudinal study as monitoring is embedded in N&G routines. In addition, although randomization would provide a higher level of evidence, it was considered unethical to randomly assign low-SES parents to N&G+ or N&G information and impractical for such a popular website (contamination bias) [159]. Moreover, 3 typical potential sources of biases in longitudinal designs may not affect our study: the use of proportions will control for historical events, there will be no *respondent fatigue* as our 2-year pilot data showed that the monthly number of IAM raters is stable, and the social desirability bias will affect pre- and postimplementation periods in a similar manner.

Considering the commitment, expertise, and networks of our team, we expect no major challenges. This project is highly facilitated by the participatory research approach (a form of integrated knowledge translation). In addition, it is sustainable as our results will be applied and sustained by 2 longstanding organizations. N&G is fully funded since 1998 by the Lucie et André Chagnon Foundation (one of the largest philanthropic agencies in Canada) and is a priority of this foundation. AKH is owned by the Hospital for Sick Children (Toronto) and is fully funded since 2004 by multiple maternal and child health agencies.

### Conclusions

The results of this study will provide a deep understanding of how low-SES parents use online child information and interact with Web editors. Following the implementation of IAM+N&G+, results will also elucidate subsequent health outcomes for low-SES parents and children after interaction with Web editors have been optimized. Thus, our results can immediately benefit millions of information users, children’s parents and relatives in particular, with a low literacy level.

## Abbreviations

AKH: AboutKidsHealth
CDIAP: Canadian Deprivation Index Assignment Program
CIHR: Canadian Institutes of Health Research
IAM: Information Assessment Method
IRB: institutional review board
N&G: Naître et grandir
NPI: nominated principal investigator
OPR: organizational participatory research
SES: socioeconomic status
